# High-speed shaking of frozen blood clots for extraction of human and malaria parasite DNA

**DOI:** 10.1186/1475-2875-10-229

**Published:** 2011-08-08

**Authors:** Klara Lundblom, Alex Macharia, Marianne Lebbad, Adan Mohammed, Anna Färnert

**Affiliations:** 1 Infectious Diseases Unit, Karolinska Institutet, Center for Molecular Medicine, L8:01, Karolinska University Hospital, Solna, SE-171 76 Stockholm, Sweden; 2 Kenya Medical Research Institute, Centre for Geographical Medicine Research (Coast), Kilifi, Kenya; 3 Department of Parasitology, Mycology and Environmental Microbiology, Swedish Institute for Infectious Disease Control, Stockholm, Sweden

## Abstract

**Background:**

Frozen blood clots remaining after serum collection is an often disregarded source of host and pathogen DNA due to troublesome handling and suboptimal outcome.

**Methods:**

High-speed shaking of clot samples in a cell disruptor manufactured for homogenization of tissue and faecal specimens was evaluated for processing frozen blood clots for DNA extraction. The method was compared to two commercial clot protocols based on a chemical kit and centrifugation through a plastic sieve, followed by the same DNA extraction protocol. Blood clots with different levels of parasitaemia (1-1,000 p/μl) were prepared from parasite cultures to assess sensitivity of PCR detection. In addition, clots retrieved from serum samples collected within two epidemiological studies in Kenya (n = 630) were processed by high speed shaking and analysed by PCR for detection of malaria parasites and the human α-thalassaemia gene.

**Results:**

High speed shaking succeeded in fully dispersing the clots and the method generated the highest DNA yield. The level of PCR detection of *P. falciparum *parasites and the human thalassaemia gene was the same as samples optimally collected with an anticoagulant. The commercial clot protocol and centrifugation through a sieve failed to fully dissolve the clots and resulted in lower sensitivity of PCR detection.

**Conclusions:**

High speed shaking was a simple and efficacious method for homogenizing frozen blood clots before DNA purification and resulted in PCR templates of high quality both from humans and malaria parasites. This novel method enables genetic studies from stored blood clots.

## Background

Efficient nucleic acid purification is a prerequisite for high quality DNA and high sensitivity of downstream molecular analyses. Under optimal conditions, sampling of blood for genetic assessments includes addition of an anticoagulant to facilitate the extraction process. Genetic analyses are, however, not always initially planned and sampling procedures thus not specifically adjusted; such as serum samples collected for antibody assays. The clots retrieved from such samples are a highly valuable source of human and pathogen DNA but are often disregarded due to troublesome handling.

Different mechanical and chemical protocols have been suggested for extraction of DNA from clots [1-8]. Nonetheless, blood clots might provide insufficient quality and quantity of DNA leading to incomplete data sets [2-4] especially after long-term storage [5]. Genetic studies targeting pathogens such as malaria parasites found in erythrocytes rely on optimal clot disruption in order to achieve high PCR sensitivity in low-density infections.

Mechanical preparation of frozen blood clots by manual crushing or cutting is inconvenient and exposes the operator to the hazard of direct contact with potentially infected blood [6-8]. Moreover, extensive manual handling involves a risk of cross-contamination between samples.

A new method for processing clots for DNA extraction using high-speed shaking in a cell disruptor was identified. The technique is based on a linear top-to-bottom shaking of the sample tube at 3,450 oscillations/minute. The method was compared with two commercially available methods: a chemical protocol and centrifugation through a plastic sieve. The success of the subsequent DNA extraction after the respective clot dispersion steps was evaluated by measuring DNA yield as well by the success of PCR detection of malaria parasites and the human α-thalassaemia gene.

## Methods

### Materials

Blood clots (n = 15) were prepared by mixing non-parasitized whole blood from a healthy Swedish donor with *in vitro *culture of the 3D7 *Plasmodium falciparum *laboratory line in 10- fold dilutions (corresponding to 1-1,000 parasites/microlitre). The tubes were centrifuged 15 minutes at 4,000 rpm and the serum was discarded. The remaining blood clot samples were stored in 2 ml apex tubes at -80°C for one month.

Serum samples were collected within two epidemiological studies (n = 588 and 316 samples respectively) in Kilifi, an area of low-moderate malaria transmission on the coast of Kenya [9]. In both studies, venous blood (2 ml) was collected in a Microtainer tube (Becton Dickinson) containing clot activator gel without anticoagulant. The tubes were centrifuged one minute at 3,000 rpm with the lid facing downwards, resulting in transfer of the activator gel to the bottom of the tube. Serum and blood clots were then separated and stored separately in 2 ml apex tubes at -80°C. One study involved children (three months -two years old) participating in a birth cohort with clot samples (100-1,000 μl) stored 2-8 years. Consent was obtained through parents or guardians of the study participants. The second study included 100-200 μl clot samples collected one month prior to extraction. Written informed consent was obtained. Thick and thin films collected at the same time as the blood samples were stained with Giemsa and analysed by conventional microscopy by two readers in order to establish the presence and density of Plasmodium parasites.

### Clot dispersion

Three methods for clot dispersion were evaluated:

1) *Chemical protocol for blood clots: *The *PG04 *protocol^® ^*(Qiagen) *is based on the Puregene Blood protocol for Gentra^® ^Puregene^® ^Blood Kit (Qiagen) but with a ten-fold increase of reagent volumes in all steps except for a three-fold increase of protein precipitation solution; moreover, a DNA-carrier was included and the incubation time with cell lysis buffer was prolonged from 10 seconds to overnight, all according to the manufacturer's instructions.

2) *Centrifugation through a plastic sieve*: The blood clots were transferred into individual plastic sieves (Clotspin^® ^Baskets, Qiagen) inserted into 50 ml tubes. The tubes were centrifuged at 2,000 g for 5 minutes forcing the clot material through the sieve to the bottom of the tubes.

3) *High speed shaking: *After addition of 1 ml lysis buffer (RBC lysis buffer, Qiagen) the original tubes were placed in a cell disruptor (Mini Bead Beater^®^, Biospec) and shaken for different length of time (10 seconds, 40 seconds, 2 minutes, respectively). The beads, recommended by the manufacturer for homogenization of tissues, were not added to minimize handling. The method is demonstrated in the Supplementary video (Additional file 1).

### DNA extraction

After clot dispersion by the respective methods, DNA was extracted with the Gentra^® ^Puregene^® ^Blood Kit (Qiagen) and addition of Proteinase K following the manufacturers protocol for whole blood. Proteinase K (20 mg/μl, 60 μl/sample) was added to the cell lysis buffer and samples were incubated at 37°C for 2 hours. DNA was dissolved in a final volume of 150 μl.

### DNA quantification

The DNA concentration (ng/μl) in 1 μl DNA suspension was measured by spectrophotometry (NanoDrop 1000, Thermo Scientific) in duplicate measurements.

### PCR detection of malaria parasites

A nested PCR method targeting the single copy gene encoding the merozoite surface protein 2 (*msp2*) was performed to detect *P. falciparum *parasites [10]. DNA prepared from in vitro cultured parasites were analysed in duplicate in two separate PCR reactions and products were visualized under UV light after electrophoresis on 2% agarose gels. In the field study in which parasite genotyping was performed, PCR products were analysed by capillary electrophoresis [11]. The lengths of *msp2 *fragments range between 250-700 bp.

### PCR analysis of the human thalassaemia gene

The α-3.7 thalassaemia-determinants and the control gene *LIS1 *were analysed by a multiplex nested PCR method followed by fragment analysis with gel electrophoresis [12]. A total of three primer pairs flanking the deletion were used in the reaction, resulting in the amplification of a 2,100 bp fragment in the absence of a deletion and a 1,800 bp product in the presence of the 3.7 deletion. Moreover, two primers encoding the housekeeping gene *LIS 1 *were included and resulted in amplification of 2,500 bp fragments.

## Results

### Dispersion of laboratory prepared clot

The three methods for clot dispersion were compared for their ability to homogenize clots before DNA extraction by the same protocol. Thereafter, the DNA yield and sensitivity of PCR detection of malaria parasites was assessed in clot samples with different parasite densities.

The chemical method following the PG04 protocol (Qiagen) failed to disperse the clots, despite overnight incubation in cell lysis buffer as recommended by the manufacturer. Only the outer part of the clot was dissolved, with a large core remaining unaffected.

Centrifugation of the clot through a plastic sieve (Clotspin^® ^Basket, Qiagen) disrupted parts of the clots, however, clot particles were stuck in the sieve and had to be transferred manually to the bottom of the tubes. Remaining material was troublesome to fully pipette out from the sieve.

High speed shaking succeeded in efficiently dispersing the clots. After shaking the original tube 40 seconds, the blood clots were totally homogenized in the lysis buffer (Supplementary video). The product was easily pipetted, consisting of mainly dissolved material. Occasionally small fragments remained but these were dissolved in the following cell lysis step. A 10 seconds shaking did not consistently break up the clots, whereas shaking for two minutes caused heating of the samples.

The degree of clot dispersion correlated to the success of PCR detection of *P. falciparum *parasites. Processing by high-speed shaking resulted in consistent detection of parasites in all clot samples in dilutions down to 10 infected erythrocytes/μl (Table 1). The PG04 and Clotspin Basket methods, however, generated consistent detection only in samples with 1000 parasites/μl, respectively, and inconsistent or negative results at lower parasite densities.

**Table 1:** Sensitivity of PCR detection of malaria parasites in clots prepared with different parasite densities processed with three methods for clot dispersion

	Method for clot disruption					
Parasite density (parasites/μl)	Chemical PG04		**Clotspin**^**® **^**Basket**		High-speed shaking	
	Nr PCR positive runs	Yield (ng/ul)	Nr PCR positive runs	Yield (ng/ul)	Nr PCR positive runs	Yield (ng/ul)
**1000**	5/5	1.0	5/5	2.2	5/5	26.4
**100**	1/5	0.7	2/5	3.3	5/5	11.5
**50**	2/5	3.5	1/5	2.7	5/5	2.4
**10**	0/5	0.3	1/5	5.5	4/5	2.5
**1**	0/5	1.5	0/5	1.9	0/5	ND

The DNA yield was overall highest in samples processed by high speed shaking, although this could not be demonstrated statistically. Despite low yield (< 10 ng/μl) in clot samples with 10 parasites/μl, PCR detection of parasites was successful after high-speed shaking. Some samples processed with the two other methods were PCR negative despite having similar or higher DNA yields (Table 1).

### Evaluation of high speed shaking in field samples

Blood clot samples (n = 588) from asymptomatic children 0-2 years in Kenya, were homogenized by high speed shaking before DNA extraction with Puregene Blood protocol (Qiagen). The level of detection of *P. falciparum *parasites was higher by PCR than by microscopy. In addition to the 25 samples positive by microscopy, parasites were detected by PCR in another 27 microscopy negative samples, resulting in a parasite prevalence of 4.3% by microscopy and 8.8% by PCR, respectively.

**Figure 1:**
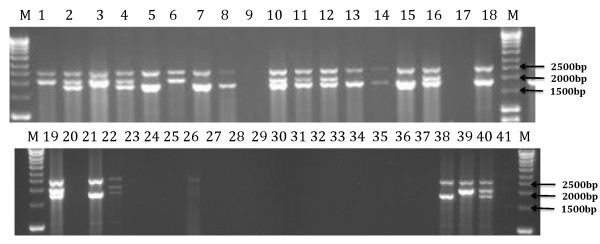
**Genotyping of the α-thalassaemia gene in blood clot samples from Kilifi District Kenya processed with high speed shaking and vortexing**. Agarose gel showing PCR amplicons of different sizes used in the diagnosis of α-3.7 thalassaemia. The heaviest amplicon is a 2.5 kb fragment representing the *lis 1 *gene, which is used as a control for amplification success. The lanes M represents the molecular weight ladder; 1-18, amplification of clot DNA extracted using the high speed shaking method; 19-37, amplification of clot DNA extracted using the vortexing method; 38-40, positive controls; 41; DNA negative control.

Genotyping of the human thalassaemia gene in Kenyan donors (n = 316) was performed in clot samples processed by high speed shaking as described above. The common African -3.7 kb α-globin deletion was successfully genotyped in 290 samples (92%). These were compared to 48 clot samples from the same study processed by vortexing at 2800 rpm for 2 min before Puregene extraction. The success rate in amplification of these samples after vortexing was 27% (Figure 1).

## Discussion

Blood clots are a valuable source of human and parasite DNA. Homogenization of the clot is, however, a prerequisite for optimal extraction of DNA from the whole sample. High speed shaking in a cell disruptor was identified as a new method for processing blood clots before DNA extraction. The method was fast and simple with minimal manual handling, limiting the risks of cross-contamination between samples. Addition of beads, recommended by the manufacturer for homogenizing tissue and faecal samples, was not needed to achieve clot dispersion and this step was therefore omitted in order to increase throughput and minimize the risk of losing material.

High speed shaking was more efficacious than two commercial kits manufactured for DNA extraction from blood clots; a chemical protocol and centrifugation through a sieve. This was reflected both by the degree of homogenization as well as the level of PCR detection. By analyzing clots prepared with different known parasite densities the threshold of detection was established in samples with low level of parasites. Samples processed with high-speed shaking achieved the highest sensitivity and the same level of PCR detection, i.e. 10 parasites/μl blood, as when blood is collected under optimal conditions with an anticoagulant eg. EDTA.

The DNA yield was similar in several samples despite processing by different methods and higher in some samples processed by high speed shaking. The sensitivity of PCR detection, however, appeared to be more informative for evaluation of the success of DNA purification. Samples with low DNA concentrations, not expected to generate positive PCR, were positive by PCR. Whereas other samples with higher yields resulted in negative PCR.

Detection of malaria parasites in clot samples collected in clinical studies in a malaria endemic area and processed by high speed shaking generated higher level of detection by PCR compared to microscopy. This is in line with our previous experience when blood is collected in EDTA. A limitation of the current study was that a systematic assessment of different clot protocols in field samples could not be performed since there were no additional clots from the same individuals for comparison.

Typing of the thalassaemia gene after shaking resulted in a detection level (92%), which is comparable to that of EDTA blood in this setting.

Optimally 100% of the samples processed by high speed shaking should become PCR positive in the thalassaemia genotyping, however the yield is likely to vary depending on the downstream method used in DNA isolation upon high speed shaking. The PCR detection was as low as 27% among a set of samples extracted using the standard Puregene protocol, including vortexing, for DNA extraction. Both methods shake the samples at high frequency 2,500-3,200 and 2,800 rpm, respectively, however high speed shaking provides a much more forceful agitation and thus more efficient disruption of the clot. A concern with mechanical disruption is the risk of shearing long DNA fragments. In this study, genes up to 2,500 bp length were successfully amplified, suggesting that the DNA was not markedly fragmented.

High speed shaking is a very simple method that requires only the mechanical device. The high noise when the machine is in use should be considered when choosing location in the laboratory. The efficient homogenization achieved by shaking allows for aliquoting of clot samples, which is otherwise troublesome, and suggests that quantitative PCR might be performed on clot samples. Here, the shaking was performed prior to extraction with the commercial Puregene kit (Qiagen). It is, however, likely that it will be equally useful together with other methods for DNA purification.

## Conclusions

High speed shaking of blood clots in a cell disruptor, without addition of beads, successfully homogenized clots and generated high quality of DNA with PCR detection levels similar to what is achieved from whole blood collected in EDTA. This novel method is a highly useful step in the extraction of human and pathogen DNA from blood clots retrieved from serum samples.

## Abbreviations

*msp 2*: merozoite surface protein 2 gene; p/μl: number of parasite infected erythrocytes per microliter blood; PCR: polymerase chain reaction; EDTA: ethylene-ediamine tetra acetic acid; LIS1: lissencephaly gene 1; bp: base pairs; rpm: revolutions per minute.

## Competing interests

The authors declare that they have no competing interests.

## Authors' contributions

KL participated in study design, carried out lab work and drafted the manuscript. AM and AM performed human molecular genetic studies and helped to write the manuscript. ML invented the idea of using high-speed shaking, helped with study design and manuscript writing. AF participated in study design, interpretation of data and manuscript writing. All authors read and approved the final manuscript.

## Supplementary Material

Additional file 1 **Video demonstrating high-speed shaking of blood clots**. Please note that in the video, the cell disruptor is started without the safety guard down which does not meet laboratory safety standards. This was only done to allow visualization of the device in use. Click here for file
